# Urinary hyaluronidase activity is closely related to vasopressinergic system following an oral water load in men: a potential role in blood pressure regulation and early stages of hypertension development

**DOI:** 10.3389/fendo.2024.1346082

**Published:** 2024-06-25

**Authors:** Anna Calvi, Alice Bongrani, Ignazio Verzicco, Giuliano Figus, Vanni Vicini, Pietro Coghi, Alberto Montanari, Aderville Cabassi

**Affiliations:** ^1^ Clinica e Terapia Medica, Department of Medicine and Surgery, University Hospital of Parma, Parma, Italy; ^2^ Cardiorenal and Hypertension Research Unit, Department of Medicine and Surgery, University of Parma, Parma, Italy

**Keywords:** hyaluronidase, arginin-vasopressin, ADH, hypertension, Aquaporin 2, water metabolism, water load, blood pressure

## Abstract

**Introduction:**

Blood pressure (BP) regulation is a complex process involving several factors, among which water-sodium balance holds a prominent place. Arginin-vasopressin (AVP), a key player in water metabolism, has been evoked in hypertension development since the 1980s, but, to date, the matter is still controversial. Hyaluronic acid metabolism has been reported to be involved in renal water management, and AVP appears to increase hyaluronidase activity resulting in decreased high-molecular-weight hyaluronan content in the renal interstitium, facilitating water reabsorption in collecting ducts. Hence, our aim was to evaluate urinary hyaluronidase activity in response to an oral water load in hypertensive patients (HT, n=21) compared to normotensive subjects with (NT+, n=36) and without (NT-, n=29) a family history of hypertension, and to study its association with BP and AVP system activation, expressed by serum copeptin levels and urine Aquaporin 2 (AQP2)/creatinine ratio.

**Methods:**

Eighty-six Caucasian men were studied. Water load test consisted in oral administration of 15–20 ml of water/kg body weight over 40–45 min. BP, heart rate, serum copeptin, urine hyaluronidase activity and AQP2 were monitored for 4 hours.

**Results:**

In response to water drinking, BP raised in all groups with a peak at 20–40 min. Baseline levels of serum copeptin, urinary hyaluronidase activity and AQP2/creatinine ratio were similar among groups and all decreased after water load, reaching their nadir at 120 min and then gradually recovering to baseline values. Significantly, a blunted reduction in serum copeptin, urinary hyaluronidase activity and AQP2/creatinine ratio was observed in NT+ compared to NT- subjects. A strong positive correlation was also found between urinary hyaluronidase activity and AQP2/creatinine ratio, and, although limited to the NT- group, both parameters were positively associated with systolic BP.

**Discussion:**

Our results demonstrate for the first time the existence in men of a close association between urinary hyaluronidase activity and vasopressinergic system and suggest that NT+ subjects have a reduced ability to respond to water loading possibly contributing to the blood volume expansion involved in early-stage hypertension. Considering these data, AVP could play a central role in BP regulation by affecting water metabolism through both hyaluronidase activity and AQP2 channel expression.

## Introduction

According to the 2023 World Health Organization (WHO) estimates, 1.28 billion adults between 30 and 79 years old suffer from hypertension and about 1 in 5 have it under control (1). Hypertension is currently the most prevalent risk factor for cardiovascular disease and one of the leading causes of premature death worldwide, with the risk being higher the younger the age of hypertension onset (2).

Blood pressure (BP) regulation is extremely complex, and several endocrine, nervous, vascular and immune factors are closely interconnected in hypertension pathogenesis (3). Water and sodium balance is undoubtedly one of them. As early as 1972, Guyton et al. demonstrated the influence of kidney in BP regulation, showing that increasing BP levels were associated with a significant increase in sodium and, consequently, water renal excretion aimed at reducing extracellular volume and restoring normal BP values (4). While sodium contribution to hypertension development has since been extensively and thoroughly studied, the role of water is still poorly defined.

Arginin-vasopressin (AVP), also known as antidiuretic hormone (ADH), is a 9-amino-acid peptide synthetized by the magnocellular neurons located in the supraoptic and paraventricular nuclei of hypothalamus and secreted in response to raised serum tonicity and/or reduced effective circulating arterial blood volume (5). Characteristically, AVP acts as an antidiuretic hormone by activating V2 receptors on the basolateral membrane of the principal cells in the collecting ducts. This is followed by Aquaporin 2 (AQP2) translocation to the luminal membrane and the formation of AQP2 channels, resulting in increased water permeability, water reabsorption and, finally, excretion of hyperosmotic urine (5). A possible role for AVP in hypertension development has been evoked since the 1980s (6–10), but, to date, is still controversial (11–13). Indeed, AVP was first identified as a pressor hormone (14), and, *in vitro*, its vasoconstrictor effect, mediated by V1a receptors on smooth muscle cells, has shown to be greater than that of angiotensin II (15, 16). *In vivo*, however, its importance is presumably limited by the activation of counter-regulatory mechanisms, including the baroreceptor reflex and the modulation of cardiac output by AVP itself (15, 16).

Interestingly, in 1958, Ginetzinsky demonstrated that hyaluronic acid (HA) metabolism in the renal interstitium is closely related to water handling and AVP action (17). Indeed, HA content in renal medullary significantly influences the process of urine concentration and dilution, mainly by altering the interstitial osmotic gradient that draws AVP-induced water reabsorption at the collecting duct level (18, 19). In humans, the degradation of high-molecular-weight HA into low-molecular-weight fragments is catalyzed by six different types of hyaluronidases, of which HYAL1 and HYAL2 are the most highly expressed and actively involved in HA metabolism (20, 21). Their activity is tightly regulated by several factors, including hormones involved in water balance, such as angiotensin II and AVP (22). Notably, AVP seems to accelerate HA catabolism in renal medullary by increasing both hyaluronidase synthesis and activity (23).

As hyaluronidase excretion in the urine has been shown to be inversely correlated to body water balance (19), the aim of our study was to assess urinary hyaluronidase activity and to study its association with BP and vasopressinergic system activation in response to an oral water load. Hypertensive patients were compared to normotensive men with and without a family history of hypertension. Since over 90% of circulating AVP is bound to platelets and its plasma half-life is only 5 to 20 min (24), the urinary AQP2/creatinine ratio, which reflects the shedding phenomenon of AQP2 from the apical luminal membrane of collecting duct principal cells, has been considered as valid surrogate for assessing renal sensitivity to AVP action, based on results obtained in a rat model of essential hypertension (25). To further confirm our results, we also assessed serum levels of copeptin, the 39-aminoacid C-terminal part of the AVP precursor (pre-proAVP), which is secreted into circulation in equimolar concentration with AVP, thus indicating AVP plasma levels (24).

## Materials and methods

### Study population

Recruitment of study population took place between 2005 and 2008 as part of the I Demand (Italy Developing Education and awareness on Micro-Albuminuria in patients with hypertensive Disease) Project, a multicenter observational study promoted by the Italian Society of Hypertension. The study was conducted in accordance with the Declaration of Helsinki principles and was approved by the Ethics Committee of the University Hospital of Parma. All participants gave written informed consent.

Included subjects referred to the Hypertension and Cardio-Renal Disease Study Center attached to the Internal Medicine Department of the University Hospital of Parma with a diagnosis of essential hypertension never treated with antihypertensive drugs (HT group, n=21). Secondary hypertension was, however, excluded by history, physical examination, blood and urine hormone tests, and renal artery ultrasound. Controls were untreated normotensive healthy volunteers mainly recruited among healthcare professionals and students. Men with both parents suffering from arterial hypertension were classified as normotensive positive subjects (family history of hypertension, NT+, n=36); otherwise, they were considered normotensive negative controls (NT-, n=29).

All subjects were men of Caucasian ethnicity. Women were excluded to avoid potential bias due to the well-known effects of estrogens on the physiological regulation of water-sodium balance, especially on AVP system activation (26). Other exclusion criteria included:

- Age < 18 years
- Ongoing urinary infection
- Heart failure
- Diabetes mellitus or insipidus
- Oedema
- Liver diseases
- Systemic connective tissue diseases.

### Water load test

Oral water load and related analyses were performed in the Day Hospital ward of the Internal Medicine Department at eight o’clock a.m. The water load experimental protocol by Velazquez et al. (27) was applied. Subjects were instructed to follow a 7-day diet containing sodium 153 mEq/day and potassium 75 mEq/day and to abstain from smoking and drinking coffee or tea for at least 12 hours before the test. Sodium and potassium levels were verified in 24-hour urine at both 72 and 24 hours prior to the test: levels above or below 25% of the expected values were considered as a protocol violation and subjects were excluded. A fast from solids starting at midnight the previous evening was also required. Small amounts of water were allowed.

Water load test consisted in oral administration of 15–20 mL of water/kg of body weight over 40–45 minutes, followed by supine rest for at least 4 hours. Systolic and diastolic office blood pressure (SBP and DBP, respectively), as well as heart rate, were assessed by the ward nurse using the cuff method every 20 minutes during the first hour and then every 30 minutes up to 4 hours. Body weight and height, as well as 24h-urine volume, were recorded prior to the water load test.

### Biological sampling

Venous blood and urine samples were obtained after three series of BP and heart rate measurements, centrifuged and immediately frozen at -80°C for subsequent analysis.

Blood was drawn prior to the test, after at least 30 minutes of supine rest, and collected into propylene tubes containing 1.5 mg/mL EDTA (ethylenediaminetetraacetic acid) buffer. Plasma sodium, potassium, osmolarity and creatinine levels were assessed using standard techniques. Serum copeptin was measured by a commercial enzyme-linked immunosorbent assay (ELISA) kit according to manufacturer’s instructions (Human Copeptin ELISA kit CSB-E121304, Cusabio Biotech, China). Intra-assay and inter-assay coefficients of variation were <8% and <10%, respectively. Analysis was performed in duplicate.

Urine samples were collected at baseline, then every 20 minutes during the first hour, every 30 minutes up to 180 minutes, and finally at 240 minutes.

### Hyaluronidase activity and AQP2/creatinine ratio assessment

Hyaluronidase activity was quantified in urine by turbidimetric assay, determining turbidity caused by the high-molecular-mass (>6–8 kDa) residual substrate precipitated with cetyltrimethylammonium bromide. The incubation mixture contained citrate-phosphate buffer (solution A: 0.1 M Na_2_HPO_4_, 0.1 M NaCl; solution B: 0.1 M citric acid, 0.1 M NaCl; solutions A and B were mixed in the appropriate proportions to achieve a 5.0 pH), 30 μL of BSA solution (0.2 mg/mL in water), 30 μL of HA substrate solution (2 mg/mL in water), 50 μL of H_2_O, 10 μL of Me_2_SO and 30 μL of enzyme solution. After 30-minute incubation at 37°C, 720 μL of 2.5% (w/v) cetyltrimethylammonium bromide solution (2.5 g of cetyltrimethylammonium bromide dissolved in 100 mL of 0.5M sodium hydroxide solution at pH 12.5) was added in order to precipitate the residual high-molecular-mass substrate and stop the enzymatic reaction. The mixture was then incubated a second time at 25°C for 20 minutes and the turbidity of each sample was determined at 600 nm with a spectrophotometer. The obtained hyaluronidase activity levels were then corrected for urinary creatinine concentration. Measurements were performed in triplicates according to Botzki et al. (28).

AQP2 levels were assessed in urine using a commercial ELISA kit (SEA580Hu 96, intra- and inter-assay coefficients of variation <10% and <12%, respectively, Cloud-Clone Corp., Katy, TX77494, USA) and then normalized for urinary creatinine concentration.

All laboratory assays were performed without freeze-thaw cycles of the samples and by investigators blind to clinical data.

### Statistical analysis

Results are expressed as mean ±standard deviation (SD).

Prior to analysis, the normal distribution of data was verified with the Shapiro-Wilk and Kolmogorov-Smirnov tests, and, when necessary, the logarithmic transformation was applied. Comparisons among groups were then made using one-way ANOVA followed by Bonferroni’s *post-hoc* test or two-way ANOVA with repeated measures followed by Dunnett’s *post-hoc* test, as appropriate. As concerns serum copeptin levels, due to the great variability of the values obtained, data were analyzed as percent changes from baseline by employing a mixed-effect model followed by Dunnett’s *post-hoc* test. The correlations between SBP and urinary hyaluronidase activity and AQP2/creatinine ratio, respectively, were evaluated by single linear regression.

Statistical analysis was performed using GraphPad Prism software (version 10.0.3, San Diego, CA, USA) and p<0.05 was considered the statistical threshold to declare significance.

## Results

Anthropometric, clinical and biological features of study population are detailed in **Table 1**. A total of 86 men aged 20 to 52 years were included. HT patients were older than NT subjects (p<0.0001 versus both NT- and NT+) and, as expected, had higher baseline SBP and DBP values (p<0.0001 for both). No difference in SBP and DBP was found between NT- and NT+ men. Likewise, baseline heart rate was similar among the groups. Subjects had normal renal function and no electrolyte disturbances. After a follow-up period of 8 to 11 years from the water load test, sustained arterial hypertension was diagnosed in 4 out of 29 (14%) NT- versus 11 out of 36 (30%) NT+ men.

**Table 1:** Anthropometric, clinical and biological characteristics of study population.

	NT- (n=29)	NT+ (n=36)	HT (n=21)
**Age** (years)	27 ± 4	27 ± 5	44 ± 5****
**BMI** (kg/m^2^)	25.9 ± 2.2	25.8 ± 2.3	26.2 ± 2.8
**SBP** (mmHg)	121 ± 9	118 ± 8	154 ± 8****
**DBP** (mmHg)	77 ± 5	76 ± 6	95 ± 5****
**Heart Rate** (bpm)	74 ± 7	74 ± 8	77 ± 10
**24h-Urine Volume** (mL)	1660 ± 389	1510 ± 298	1634 ± 382
**Creatinine** (mg/dL)	0.88 ± 0.12	0.90 ± 0.15	0.92 ± 0.19
**Sodium** (mmol/L)	138 ± 3	139 ± 4	139 ± 4
**Potassium** (mmol/L)	4.4 ± 0.6	4.6 ± 0.5	4.3 ± 0.7

The values are expressed as mean ± standard deviation. NT-, Normotensive subjects; NT+, Normotensive subjects with family history of hypertension; HT, Hypertensive subjects; BMI, Body Mass Index; SBP, Systolic Blood Pressure; DBP, Diastolic Blood Pressure.

****p<0.0001 vs NT- and NT+ groups, One-way ANOVA followed by Bonferroni *post-hoc* test.

**Figure 1:**
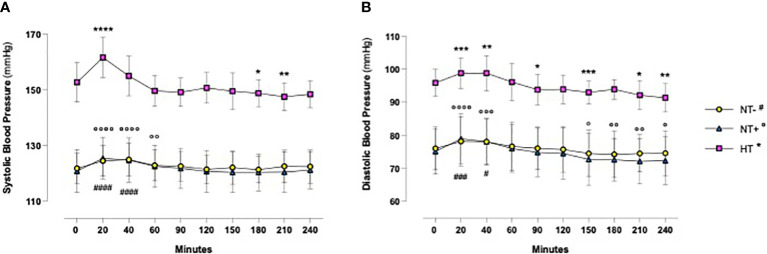
Blood pressure time course in response to oral water loading. In response to oral water loading, systolic **(A)** and diastolic **(B)** blood pressure increased rapidly with maximum values at 20 to 60 minutes and then reverted to basal or lower levels. Although hypertensive patients (HT) maintained higher BP values throughout the test (p<0.0001), this trend was similar in the three groups. *p<0.05, **p<0.01, ***p<0.001, ****p<0.0001 versus T0 for HT group; °p<0.05, °°p<0.01, °°°p<0.001, °°°°p<0.0001 versus T0 for normotensive subjects with family history of hypertension (NT+); ^#^p<0.05, ^###^p<0.001, ^####^p<0.0001 versus T0 for the normotensive group (NT-); two-way ANOVA with repeated measures followed by Dunnett’s *post-hoc* test.

**Figure 2:**
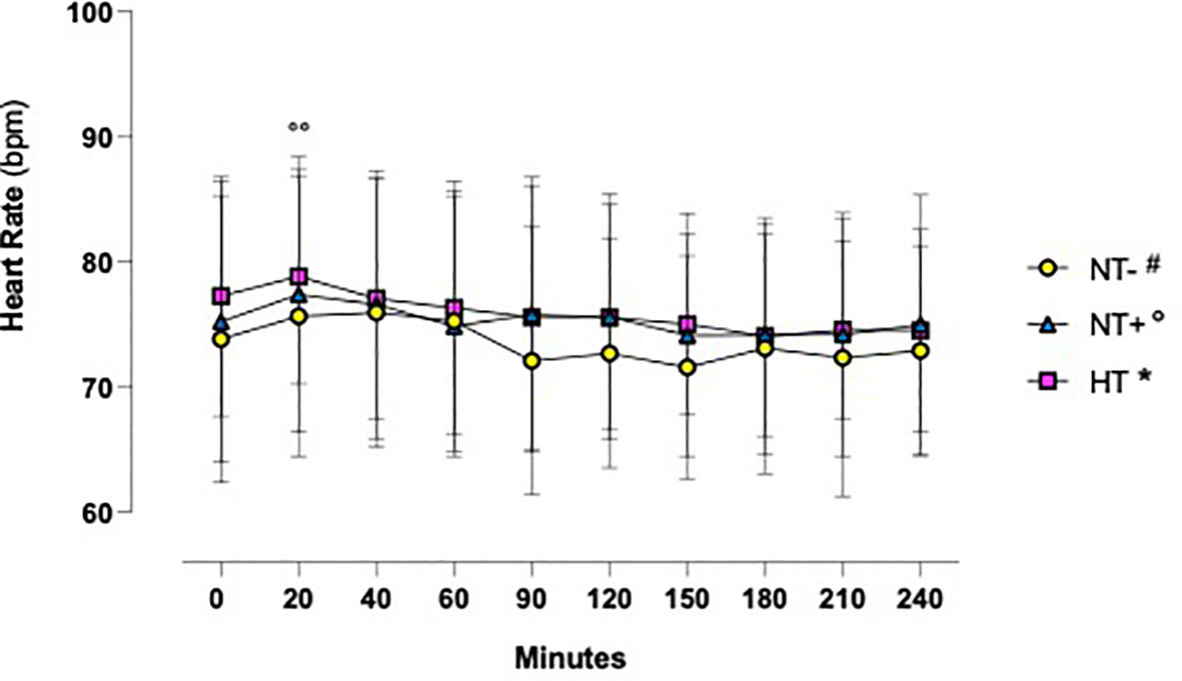
Heart rate time course in response to oral water loading. Heart Rate was not significantly influenced by oral water loading, except for a modest increase at 20 minutes in normotensive subjects with a family history of hypertension (NT+). °°p<0.01 versus T0 for NT+ group; two-way ANOVA with repeated measures followed by Dunnett’s *post-hoc* test. NT-, normotensive subjects; HT, hypertensive patients.

**Figure 3:**
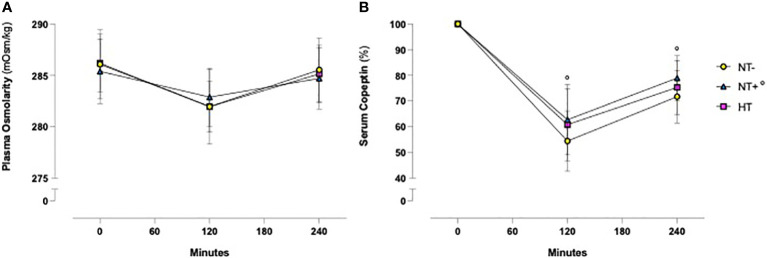
Time course of plasma osmolarity and serum copeptin levels in response to an oral water load. Oral water load resulted in a significant decrease in both plasma osmolarity **(A)** and serum copeptin **(B)** levels (p<0.0001 compared to baseline for all groups). No differences in plasma osmolarity were observed among the groups. By contrast, at 120 minutes the water load-induced reduction in serum copeptin, expressed as a percent change from baseline, was significantly lower in subjects with a family history of hypertension (NT+). °p<0.05 versus NT- group; two-way ANOVA **(A)** and mixed-effect model **(B)** with repeated measures followed by Dunnett’s *post-hoc* test. NT-, normotensive subjects; HT, hypertensive patients.

As shown in **Figure 1**, in response to oral water loading, SBP and DBP increased rapidly, with peak values at 20–40 min, and then reverted to basal or even lower levels. Although HT patients maintained higher BP values throughout the test (p<0.0001 versus normotensive subjects), this trend was similar in the three groups. By contrast, except for an early increase in NT+ men, heart rate was not significantly affected by the oral water load (**Figure 2**). No differences in baseline plasma osmolarity, as well as serum copeptin levels, were observed among the groups. However, as expected, water load resulted in a significant reduction in both parameters (p<0.001 compared to baseline for all groups, **Figures 3A, B**). Notably, at 120 minutes, while plasma osmolarity was similar among groups, NT+ subjects showed a blunted decrease in serum copeptin levels (p<0.05 versus NT- group).

**Figure 4:**
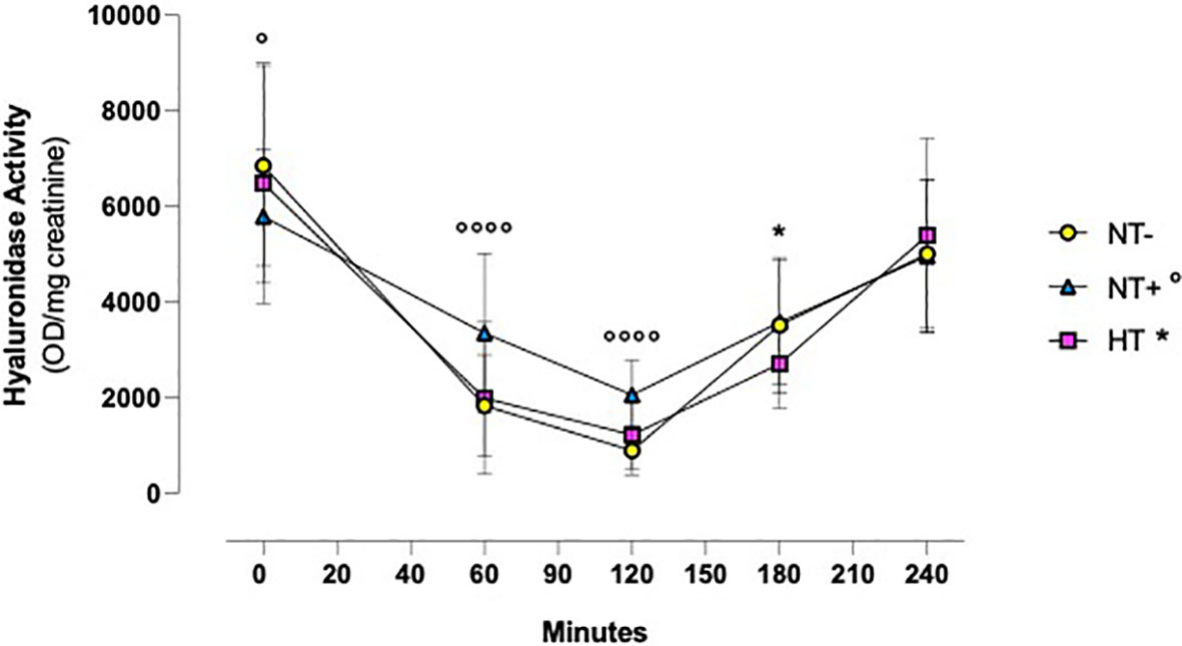
Urinary hyaluronidase activity following oral water loading. Urinary hyaluronidase activity, assessed by turbidimetric assay, was significantly reduced by water drinking, with minimal levels at 120 minutes (p<0.0001 compared to baseline for all groups) and then gradually recovered to baseline values (compared to baseline, p<0.01 for hypertensive patients (HT), p<0.001 for normotensive subjects). Although lower at T0, at 60 and 120 minutes, hyaluronidase activity levels were higher in normotensive subjects with family history of hypertension (NT+) compared to the normotensive ones (NT-). *p<0.05, HT versus NT- subjects; °p<0.05, °°°°p<0.0001, NT+ versus NT- subjects; two-way ANOVA with repeated measures followed by Dunnett’s *post-hoc* test.

Similarly, as illustrated in **Figure 4**, in all groups, urinary hyaluronidase activity was significantly reduced by water load, reaching the lowest levels at 120 min (p<0.0001 versus baseline for all groups) and then gradually recovering to baseline values (p<0.01 versus baseline for HT patients, p<0.001 for NT- and NT+ subjects). In addition, while the trends over time of NT- and HT groups were overlapping, NT+ men showed significantly higher values at both 60 and 120 min after water loading (p<0.0001 versus NT-).

**Figure 5:**
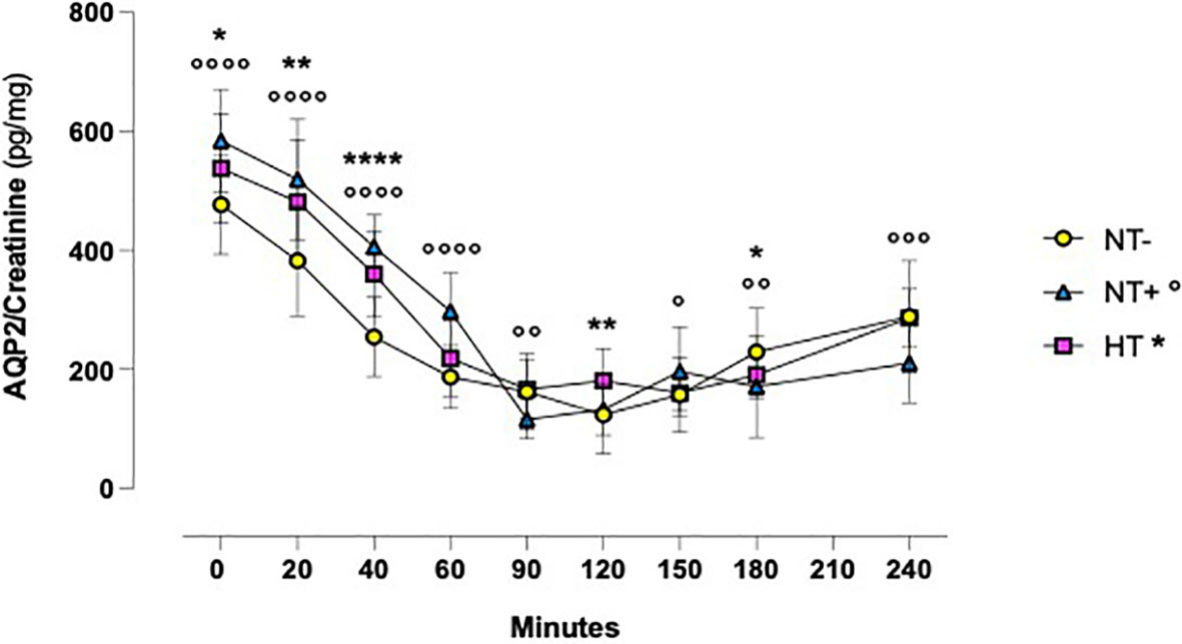
Aquaporln2 (AQP2)/creatinine ratio in response to oral water loading. After water drinking, the AQP2/creatinine ratio rapidly decreased to reach minimum values at 90 and 120 minutes (p<0.0001 for all groups at both times). Trends over time were comparable between groups, but hypertensive patients (HT) and normotensive subjects with family history of hypertension (NT+) maintained higher values than the normotensive group (NT-). *p<0.05, **p<0.01, ****p<0.0001, HT versus NT- subjects; °p<0.05, °°p<0.01, °°°p<0.001, °°°°p<0.0001, NT+ versus NT- subjects; two-way ANOVA with repeated measures followed by Dunnett’s *post-hoc* test.

Assessing the AQP2/creatinine ratio in urine, we observed a similar time course (**Figure 5**). Indeed, after water drinking, the AQP2/creatinine ratio rapidly decreased from 20 min onward to reach minimum values at 90–120 min (p<0.0001 for all groups at both times). Again, trends over time were comparable among groups, but NT+ and HT subjects had higher values than the NT- ones, especially in the first 2 hours of the test.

**Figure 6:**
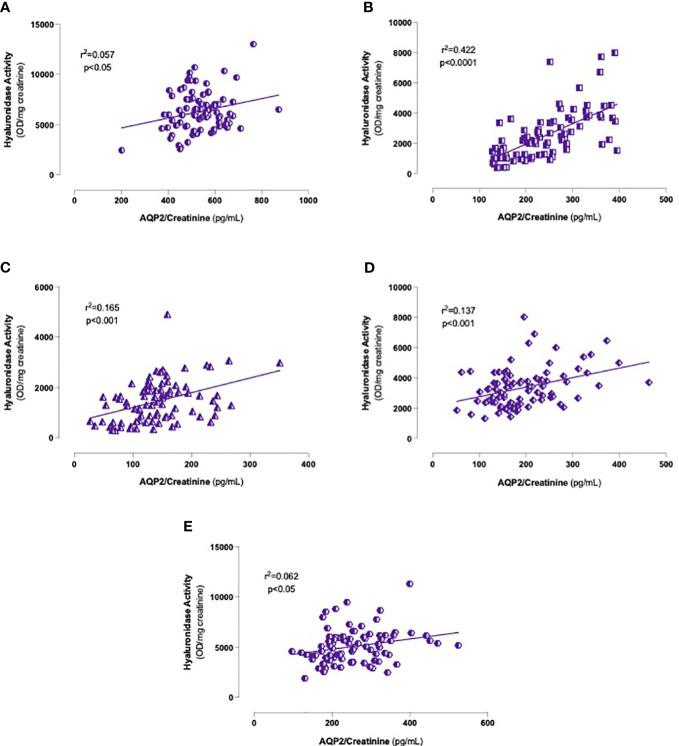
Correlations between urinary hyaluronidase activity and AQP2/Creatinine ratio levels. Onset **(A)**, 60 **(B)**, 120 **(C)**, 180 **(D)** and 240 **(E)** minutes after oral water loading. Simple linear regression.

In light of these findings, the association between urinary hyaluronidase activity and the AQP2/creatinine ratio was studied. As depicted in **Figure 6**, including all men, we found significant positive correlations at any time point, especially between 60 and 180 min after water load (**Figures 6B–D**). Interestingly, when we carried out regression analyses by groups, we observed globally consistent results, confirming the data obtained on the entire cohort (**Table 2**).

**Table 2:** Correlations between urinary hyaluronidase activity and AQP2/creatinine ratio.

Minutes	NT- (n=29)	NT+ (n=36)	HT (n=21)
**0**	r^2^ = 0.137*	r^2^ = 0.070	r^2^ = 0.332**
**60**	r^2^ = 0.341***	r^2^ = 0.291***	r^2^ = 0.354**
**120**	r^2^ = 0.326**	r^2^ = 0.471****	r^2^ = 0.438**
**180**	r^2^ = 0248**	r^2^ = 0.150*	r^2^ = 0.051
**240**	r^2^ = 0.106	r^2^ = 0.059	r^2^ = 0.125

Hyaluronidase activity and AQP2/creatinine ratio were measured in urine every 60 minutes after water loading test by turbidimetric assay and ELISA, respectively. All correlations are positive. AQP2, Aquaporin 2; NT-, Normotensive subjects; NT+, Normotensive subjects with family history of hypertension; HT, Hypertensive subjects.

*p<0.05, **p<0.01, ***p<0.001, ****p<0.0001, Simple Linear Regression.

When evaluating the associations between SBP and hyaluronidase activity, as well as AQP2/creatinine ratio, significant correlations were found only in the NT- group (**Tables 3**, **4**, respectively). In particular, while SBP was positively correlated with urinary hyaluronidase activity at baseline and at the end of the test (p<0.01 at 0 and p<0.05 at 240 min, **Table 3**), a significant positive correlation with the AQP2/creatinine ratio was detected at T0 and 40 min after water drinking (p<0.05 and p<0.01, respectively, **Table 4**), which corresponded to peak SBP levels (**Figure 1**).

**Table 3:** Correlations between SBP and urinary hyaluronidase activity.

Minutes	NT- (n=29)	NT+ (n=36)	HT (n=21)
**0**	r^2^ = 0.308 (pos)**	r^2^ = 0.002 (neg)	r^2^ = 0.010 (pos)
**60**	r^2^ = 0.003 (neg)	r^2^ = 0.017 (neg)	r^2^ = 0.003 (neg)
**120**	r^2^ = 0.04 (neg)	r^2^ = 0.004 (pos)	r^2^ = 0.000
**180**	r^2^ = 0.018 (neg)	r^2^ = 0.022 (neg)	r^2^ = 0.004 (neg)
**240**	r^2^ = 0.166 (pos)*	r^2^ = 0.004 (pos)	r^2^ = 0.013 (neg)

SBP and urinary hyaluronidase activity by turbidimetric assay were assessed every 60 minutes after water loading test. SBP, Systolic Blood Pressure; NT-, Normotensive subjects; NT+, Normotensive subjects with family history of hypertension; HT, Hypertensive subjects.

*p<0.05, **p<0.01, Simple Linear Regression.

**Table 4:** Correlations between SBP and urinary AQP2/creatinine ratio.

Minutes	NT- (n=29)	NT+ (n=36)	HT (n=21)
**0**	r^2^ = 0.175 (pos)*	r^2^ = 0.000	r^2^ = 0.044 (neg)
**20**	r^2^ = 0.103 (pos)	r^2^ = 0.004 (neg)	r^2^ = 0.018 (neg)
**40**	r^2^ = 0.241 (pos)**	r^2^ = 0.005 (neg)	r^2^ = 0.018 (neg)
**60**	r^2^ = 0.109 (pos)	r^2^ = 0.013 (neg)	r^2^ = 0.028 (neg)
**120**	r^2^ = 0.047 (neg)	r^2^ = 0.022 (neg)	r^2^ = 0.018 (neg)
**180**	r^2^ = 0.036 (neg)	r^2^ = 0.070 (neg)	r^2^ = 0.003 (pos)
**240**	r^2^ = 0.054 (pos)	r^2^ = 0.000	r^2^ = 0.077 (neg)

SBP and urinary AQP2/creatinine ratio were assessed every 20 minutes during the 1^st^ hour, then every 60 minutes until 240 minutes after water loading test. AQP2, Aquaporin 2; NT-, Normotensive subjects; NT+, Normotensive subjects with no familial history hypertension; HT, Hypertensive subjects.

*p<0.05, **p<0.01, Simple Linear Regression.

## Discussion

In our study, we first demonstrated that urinary hyaluronidase activity markedly decreases in response to an oral water load, reaching its lowest levels after 120 min. Notably, at this time, NT+ subjects showed significantly higher values than both NT- and HT men. Interestingly, the time course of serum copeptin levels and urine AQP2/creatinine ratio followed that of hyaluronidase activity with a close direct correlation. These data, along with the positive associations observed between SBP and both urinary hyaluronidase activity and AQP2/creatinine ratio, support the existence of a close relationship between renal hyaluronidases and vasopressinergic system activation, which could affect water metabolism under both normotensive and sustained hypertensive conditions.

Acute oral water intake is known to increase BP in several animal species (29) as well as in patients with orthostatic hypotension symptoms due to autonomic failure (30). In our cohort, we observed a significant and early increase in SBP and, to a lesser extent, DBP levels, which reached their highest values about 20 min after the oral water load. These findings, largely consistent with most of the literature (29, 31–34), have been reported to be associated with increased plasma norepinephrine levels, muscle sympathetic nerve activity and peripheral vascular resistance, which would be counterbalanced by vagal modulation and baroreflex activation (29, 32). In accordance with this, in response to the oral water load, we found no significant changes in heart rate, except for a modest increase in NT+ subjects at 20 min. Interestingly, SBP levels increased by 8.9 +/- 0.3 mmHg in HT group versus 4.6 +/- 0.3 mmHg and 2.7 +/- 0.3 mmHg in NT+ and NT- men, respectively, suggesting a greater sympathetic response to water drinking in HT patients. These data, confirming the results of Velasquez et al. (27), are in agreement with the finding of higher basal muscle sympathetic nerve activity in subjects with mild hypertension compared to the normotensive ones (32). In previous studies, no changes were detected in plasma volume, plasma renin activity and sodium balance (32). Furthermore, the pressor effect of drinking water did not appear to be related to water temperature or gastric distension (29) and was not obtained when pure water was replaced by salt water (32) or administered via intravenous infusion (29). Drinking water would rather activate postganglionic sympathetic neurons directly (33) or, alternatively, through a gastro-pressor reflex mediated by the osmoreceptors in the proximal gut and portal system (35, 36), triggering both sympathetic activation and reflex inhibition of AVP secretion (31). Indeed, whereas in response to oral water loading, Velasquez et al. did not show any difference in plasma AVP levels (27), Geelen et al. demonstrated a rapid decrease followed by a long-lasting inhibition of AVP secretion which was not accompanied by changes in the renin-angiotensin-aldosterone system (RAAS) (31).

Similar to what we observed in normotensive and hypertensive rats (data under submission), oral water load induced a significant decrease in urinary hyaluronidase activity with a nadir at 120 min, followed by an almost complete restoration to baseline physiological values within 4 hours. Meaningfully, NT+ men, despite having lower hyaluronidase activity prior to the test, maintained significantly higher levels than NT- subjects at both 60 and 120 minutes after water load. As far as we know, this is the first time that such a result has been described. Indeed, the effects of oral water load on hyaluronidase activity have at present been studied essentially *in vitro* (37–39) or in animal models (19, 23, 40, 41). In particular, in rats, Stridh et al. (41) demonstrated that, upon continuous hydration, medullary HA levels increased with a peak after about 2 hours and returned to control values after about 4 hours (41).

Although the inverse relationship between the renal excretion of hyaluronidase and body hydration has been known since 1958 (17, 37), the mechanism through which hyaluronidase activity influences water metabolism has not yet been fully elucidated. HA is a negatively charged, ubiquitously distributed linear glycosaminoglycan with a unique water-binding ability (42) that largely depends on its molecular weight. Indeed, when high-molecular-weight HA is above 0.2 mg/mL, inter- and intra-molecular interactions occur such that the volume occupied by HA increases, excluding other large molecules (19). This “steric exclusion” phenomenon might antagonize further water reabsorption and influence the osmotic activity in the intercellular matrix (43). Interestingly, in the kidney, the HA content is considerably higher in the inner medulla than in the cortex, where 80% of the filtered water is reabsorbed (19) and depends mainly on renal medullary interstitial cell (RMICs) activity, which is influenced by both medium osmolality and oxygen tension (38). In particular, high-molecular-weight HA, internalized in RMICs via the CD44-receptor, undergoes degradation by HYAL1 and HYAL2, which seems to be the main regulatory step determining HA content in the interstitial matrix (37, 38). A decrease in hyaluronidase activity thus results in an increase in high-molecular-weight HA content in the renal medullary, which would interfere with the urinary concentration process by modifying the kidneys’ ability to excrete or reabsorb adequate amounts of water. In addition to the “steric exclusion” effect, two other mechanisms have been proposed to explain this phenomenon. First, modification of the physicochemical characteristics of the interstitial matrix due to HA accumulation could compromise the medullary osmotic gradient. Alternatively, or complementarily, the functional oedema accompanying the increase in high-molecular-weight HA might widen the diffusion distances between tubules and blood vessels and hinder water reabsorption from the collecting ducts (18). In light of these considerations, the higher hyaluronidase activity found in NT+ subjects strongly suggests that men predisposed to develop hypertension have a lower diuretic response to water load, possibly resulting in blood volume expansion and ultimately, hypertension development.

In our study, the changes in hyaluronidase activity were accompanied by broadly comparable variations over time in serum copeptin levels and urinary AQP2/creatinine ratio, which we have previously shown to reliably reflect body water balance and vasopressinergic system activation (24, 25). Moreover, a positive correlation between urinary hyaluronidase activity and AQP2/creatinine ratio was found at any time point of the test, when analyzing both the entire cohort and each subject group separately, indicating the existence of a close relationship between hyaluronidase activity and AVP. The presence of vasopressinergic V1 (44) and V2 receptors (45) has been demonstrated in RMICs and AVP has been found to enhance both the expression and synthesis of hyaluronidases. In addition, it increases sodium and urea concentration to levels optimal for hyaluronidases activity (23) by inducing the expression of the epithelial sodium channel ENaC and the urea transporter UT-A1, respectively (5). Interestingly, with regard to animal models, the Brattleboro rats, which genetically lack AVP, show higher HA content in the renal interstitium associated with exaggerated diuresis (23), while gerbils, having 4 times higher AVP levels than rats, have 37% lower papillary HA content which could facilitate water reabsorption to ensure water conservation (40).

Significantly, prior to oral water load and 4 hours after, that is under physiological conditions in the context of a sodium- and potassium-controlled diet, urinary hyaluronidase activity was positively correlated with SBP levels, which, in turn, were positively associated with the urinary AQP2/creatinine ratio at 0 and 40 min, corresponding to BP rise in response to the oral water load. As mentioned above, the role of AVP in hypertension development has long been debated. Although patients suffering from syndrome of inappropriate ADH secretion (SIADH) have normal BP levels (11) and some authors have found no differences in plasma AVP between normotensive and hypertensive subjects (12), others have shown that individuals with hypertension have significantly higher levels of urinary and circulating AVP, which positively correlate with DBP (7, 8, 14, 27). In addition, in a variety of rat models of hypertension, including spontaneously hypertensive and DOCA-salt sensitive rats, higher levels of AVP in urine and plasma, as well as increased pressor responsiveness to AVP, have been described (6). Notably, in these animals, the antidiuretic effect of AVP appears to be necessary to make possible the expansion of circulating volume upon which these hypertensive forms depend (6). In particular, according to Share and Crofton, for AVP to contribute to hypertension, the pressor responsiveness to it must be sufficiently high, which implies both increased vascular smooth muscle cell reactivity and impaired baroreceptor activity (6).

**Figure 7:**
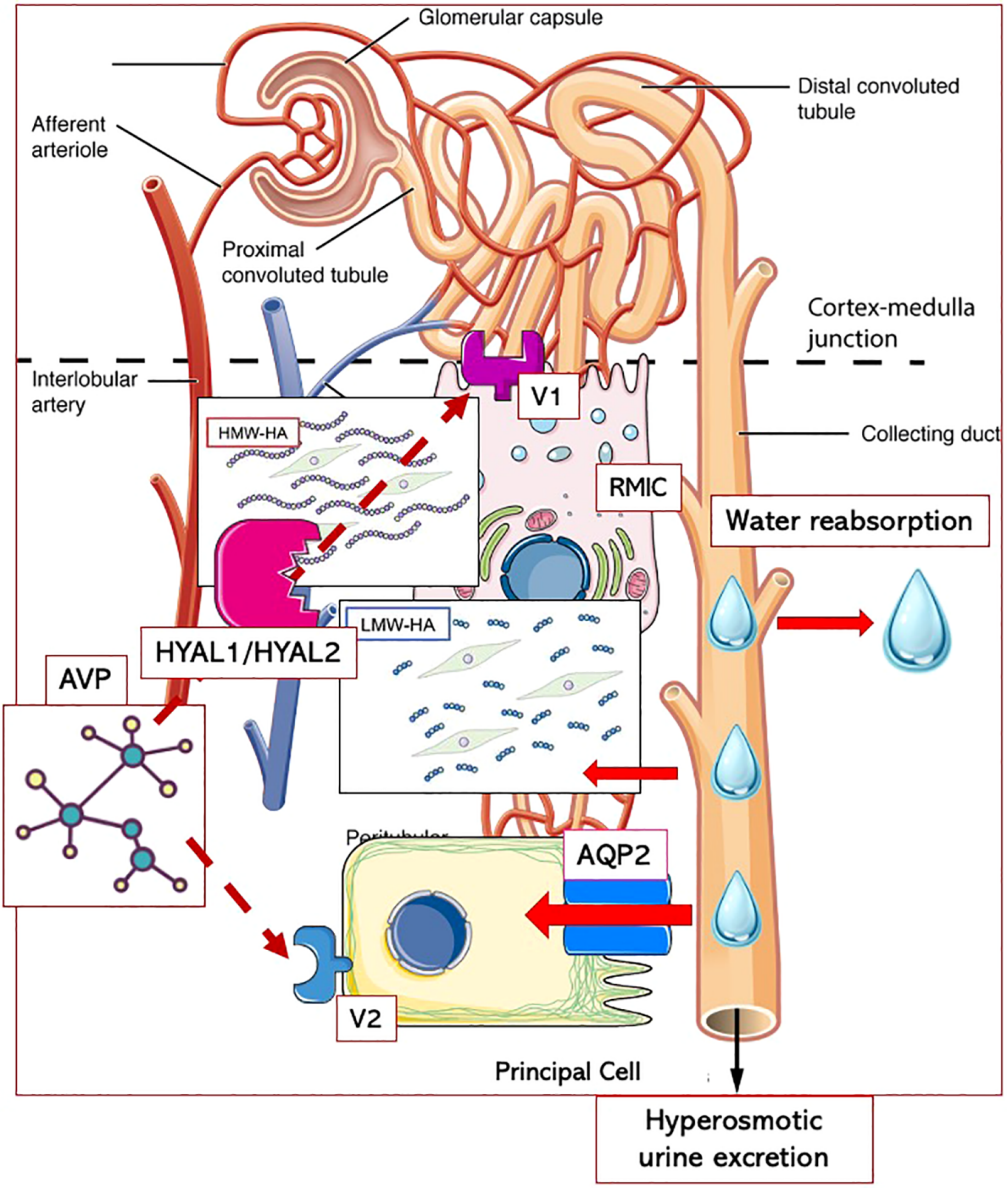
Putative interplay between AVP and hyaluronic acid metabolism in renal medullary interstitium. Arginin-Vasopressin (AVP) could act in the renal medullary through two distinct pathways: either by regulating the permeability of the apical membrane of the collecting duct principal cells through Aquaporin 2 (AQP2) translocation via V2 receptors, or by enhancing the activity of hyaluronidases (HYAL1/HYAL2) resulting in decreased content of high-molecular-weight hyaluronic acid (HMW-HA) in the interstitial matrix via V1 receptors. The degradation of HMW-HA into low-molecular-weight fragments (LMW-HA) would be essential to allow water diffusion from the tubules to the interstitium and, ultimately, to blood vessels. While hyaluronidase activity would primarily provide conditions facilitating water flow, AQP2 channels could be recruited when higher water volumes need to be moved to rapidly restore water balance. RMICs, renal medullary interstitial cells.

Thus, both the higher hyaluronidase activity levels and the increased AQP2 urinary shedding indicate that in men with a family history of hypertension, the kidneys’ ability to excrete water in response to an oral load is reduced and/or impaired. In the long-term, this altered vasopressinergic response could lead to the blood volume expansion that contributes to hypertension development in its early stages, before the sodium and RAAS contribution becomes predominant. The lack of a positive linear correlation between SBP and hyaluronidase activity, or AQP2/creatinine ratio, observed in NT+ and HT men, could be consistent with the loss of the appropriate vasopressinergic system regulation. The resulting alterations in water balance might influence BP through two distinct pathways. Indeed, as previously proposed (18, 39), AVP could act in the renal medullary either by regulating the permeability of the apical membrane of the collecting duct principal cells through AQP2 translocation via V2 receptors, or by modifying the HA content in the interstitial matrix via V1 receptors. In particular, while the role of hyaluronidase activity would be primarily to provide conditions facilitating water flow, AQP2 channels could be recruited when higher water volumes need to be moved to rapidly restore water balance (**Figure 7**).

Despite the novel and innovative findings, our study has some limitations. First, we do not currently have measurements of plasma AVP levels, urine osmolarity, or urine sodium and potassium concentrations during the water load test, which could have been important for a more complete explanation of our results. Second, hyaluronidase activity was not assessed between 0 and 60 min after the oral water load, when BP increased and the AQP2/creatinine ratio began to drop. Third, women were excluded from our study, limiting the generalization of our findings. It is, however, well-known that the incidence and severity of hypertension are lower in females than in age-matched males due to the protective effects of estrogens, which beside acting on cardiovascular and renal systems, regulate the expression and secretion of AVP by the hypothalamic paraventricular nuclei (46). Recent data obtained in rats also demonstrate that estradiol prevents salt-dependent hypertension by suppressing salt-induced GABA-ergic excitation in AVP-secreting neurons, resulting in AVP secretion that plays a crucial role in the development and maintenance of hypertension in these animals (47). To avoid any potential misleading interpretation of our results, we thus decided to exclude women, including those of postmenopausal age. Indeed, as study subjects were young men aged 20 to 52 years, the inclusion of postmenopausal women would have introduced an additional potentially confounding factor related to age. Further, our sample size is quite limited, but to the best of our knowledge, our study is the first to assess hyaluronidase activity under hyperhydration conditions in men and to evaluate its relationship with BP regulation not only in hypertensive patients, but also in normotensive subjects with a family history of hypertension. In this regard, it is noteworthy that after a follow-up period of 8–11 years from the water load test, 30% of NT+ men were diagnosed as hypertensive compared to 14% of NT- men. This does not mean that all NT+ subjects will necessarily develop this disease during their lifetime. However, since the men’s age at the follow-up ranged from 28 to 47 years, having both parents suffering from sustained hypertension is confirmed as a strong predisposing factor for the early development of arterial hypertension. Finally, since this is a noninterventional study, the associations we have pointed out allow us only to speculate about the potential role of AVP in BP regulation, but not to establish a causality link between the AVP-induced changes in HA metabolism and the predisposition to hypertension. Further confirmation of our hypotheses may come from data obtained under water deprivation conditions, which are currently being processed.

In conclusion, in our study we demonstrated for the first time the existence in men of a close association between urinary hyaluronidase activity and vasopressinergic system activation, expressed by serum copeptin levels and urinary AQP2/creatinine ratio. These findings confirm that HA handling is highly involved in renal water metabolism under the influence of AVP, which could play a key role in BP regulation under both physiological and water-load conditions by alternatively targeting hyaluronidase activity and the AQP2 channel system. With the reservation of being confirmed in larger samples, our results contribute to shed light on the complex interactions involved in the endocrine regulation of BP and highlight some pathophysiological aspects that may be deeply implicated in hypertension susceptibility, paving the way for novel potential target to act upon in the early stages of hypertension development.

## Data availability statement

The raw data supporting the conclusions of this article will be made available by the authors, without undue reservation.

## Ethics statement

The studies involving humans were approved by Ethics Committee of University Hospital of Parma. The studies were conducted in accordance with the local legislation and institutional requirements. The participants provided their written informed consent to participate in this study.

## Author contributions

AnC: Data curation, Investigation, Software, Writing – original draft, Writing – review & editing. AB: Conceptualization, Data curation, Writing – original draft, Writing – review & editing, Methodology, Software. IV: Conceptualization, Data curation, Investigation, Writing – review & editing. GF: Data curation, Investigation, Writing – original draft. VV: Data curation, Investigation, Writing – review & editing. PC: Data curation, Investigation, Writing – review & editing. AM: Conceptualization, Writing – review & editing, Methodology, Project, Resources, Supervision. AdC: Conceptualization, Data curation, Funding acquisition, Investigation, Writing – review & editing, Methodology, Project, Supervision.

## References

[1] WHO. World health organization hypertension (2023). Available online at: https://www.who.int/news-room/fact-sheets/detail/hypertension.

[2] Al Ghorani H Götzinger F Böhm M Mahfoud F. Arterial hypertension - Clinical trials update 2021. Nutr Metab Cardiovasc Dis. (2022) 32:21–31. doi: 10.1016/j.numecd.2021.09.007 34690044 PMC8444354

[3] Klimczak D Jazdzewski K Kuch M. Regulatory mechanisms in arterial hypertension: role of microRNA in pathophysiology and therapy. Blood Pressure. (2017) 26:2–8. doi: 10.3109/08037051.2016.1167355 27177042

[4] Guyton AC Coleman TG Cowley AW Scheel KW Manning RD Norman RA. Arterial pressure regulation. Am J Med. (1972) 52:584–94. doi: 10.1016/0002-9343(72)90050-2 4337474

[5] Warren AM Grossmann M Christ-Crain M Russell N. Syndrome of inappropriate antidiuresis: from pathophysiology to management. Endocrine Rev. (2023) 44:819–61. doi: 10.1210/endrev/bnad010 PMC10502587 36974717

[6] Share L Crofton JT. Contribution of vasopressin to hypertension. Hypertension. (1982) 4:III85–92. doi: 10.1161/01.HYP.4.5_Pt_2.III85 7049934

[7] Cowley AW Cushman WC Quillen EW Skelton MM Langford HG. Vasopressin elevation in essential hypertension and increased responsiveness to sodium intake. Hypertension. (1981) 3:I93–100. doi: 10.1161/01.HYP.3.3_Pt_2.I93 7262983

[8] Afsar B. Pathophysiology of copeptin in kidney disease and hypertension. Clin Hypertens. (2017) 23:13. doi: 10.1186/s40885-017-0068-y 28638629 PMC5469179

[9] Fernandes S Bruneval P Hagege A Heudes D Ghostine S Bouby N. Chronic V2 vasopressin receptor stimulation increases basal blood pressure and exacerbates deoxycorticosterone acetate-salt hypertension. Endocrinology. (2002) 143:2759–66. doi: 10.1210/endo.143.7.8918 12072411

[10] Cowley AW Szczepanska-Sadowska E Stepniakowski K Mattson D. Chronic intravenous administration of V1 arginine vasopressin agonist results in sustained hypertension. Am J Physiol. (1994) 267:H751–756. doi: 10.1152/ajpheart.1994.267.2.H751 8067431

[11] Padfield PL Brown JJ Lever AF Morton JJ Robertson JIS. Blood pressure in acute and chronic vasopressin excess: studies of Malignant hypertension and the syndrome of inappropriate antidiuretic hormone secretion. N Engl J Med. (1981) 304:1067–70. doi: 10.1056/NEJM198104303041803 7207565

[12] Sladek CD Blair ML Mangiapane M. Evidence against a pressor role for vasopressin in spontaneous hypertension. Hypertension. (1987) 9:332–8. doi: 10.1161/01.HYP.9.4.332 2951326

[13] Kawano Y. The role of vasopressin in essential hypertension plasma levels and effects of the V1 receptor antagonist OPC-21268 during different dietary sodium intakes. Am J Hypertension. (1997) 10:1240–4. doi: 10.1016/S0895-7061(97)00269-0 9397242

[14] Bankir L Bichet DG Bouby N. Vasopressin V2 receptors, ENaC, and sodium reabsorption: a risk factor for hypertension? Am J Physiol Renal Physiol. (2010) 299:F917–928. doi: 10.1152/ajprenal.00413.2010 20826569

[15] Cowley AW Monos E Guyton AC. Interaction of vasopressin and the baroreceptor reflex system in the regulation of arterial blood pressure in the dog. Circ Res. (1974) 34:505–14. doi: 10.1161/01.RES.34.4.505 4826927

[16] Montani JP Liard JF Schoun J Möhring J. Hemodynamic effects of exogenous and endogenous vasopressin at low plasma concentrations in conscious dogs. Circ Res. (1980) 47:346–55. doi: 10.1161/01.RES.47.3.346 7408117

[17] Ginetzinsky AG. Role of hyaluronidase in the re-absorption of water in renal tubules: the mechanism of action of the antidiuretic hormone. Nature. (1958) 182:1218–9. doi: 10.1038/1821218a0 13590279

[18] Stridh S Palm F Hansell P. Inhibition of hyaluronan synthesis in rats reduces renal ability to excrete fluid and electrolytes during acute hydration. Upsala J Med Sci. (2013) 118:217–21. doi: 10.3109/03009734.2013.834013 PMC4190891 24102146

[19] Hansell P Göransson V Odlind C Gerdin B Hällgren R. Hyaluronan content in the kidney in different states of body hydration. Kidney Int. (2000) 58:2061–8. doi: 10.1111/j.1523-1755.2000.00378.x 11044226

[20] Kobayashi T Chanmee T Itano N. Hyaluronan: metabolism and function. Biomolecules. (2020) 10:1525. doi: 10.3390/biom10111525 33171800 PMC7695009

[21] Fallacara A Baldini E Manfredini S Vertuani S. Hyaluronic acid in the third millennium. Polymers. (2018) 10:701. doi: 10.3390/polym10070701 30960626 PMC6403654

[22] Rügheimer L Johnsson C Maric C Hansell P. Hormonal regulation of renomedullary hyaluronan. Acta Physiol (Oxf). (2008) 193:191–8. doi: 10.1111/j.1748-1716.2007.01795.x 18081884

[23] Ivanova LN Babina AV Baturina GS Katkova LE. Effect of vasopressin on the expression of genes for key enzymes of hyaluronan turnover in Wistar Albino Glaxo and Brattleboro rat kidneys. Exp Physiol. (2013) 98:1608–19. doi: 10.1113/expphysiol.2013.073163 23955305

[24] Bolignano D Cabassi A Fiaccadori E Ghigo E Pasquali R Peracino A. Copeptin (CTproAVP), a new tool for understanding the role of vasopressin in pathophysiology. Clin Chem Lab Med (CCLM). (2014) 52(10):1447–56. doi: 10.1515/cclm-2014-0379 24940718

[25] Verzicco I Tedeschi S Graiani G Bongrani A Carnevali ML Dancelli S. Evidence for a prehypertensive water dysregulation affecting the development of hypertension: results of very early treatment of vasopressin V1 and V2 antagonism in spontaneously hypertensive rats. Front Cardiovasc Med. (2022) 9:897244. doi: 10.3389/fcvm.2022.897244 35722114 PMC9198251

[26] Sladek CD Somponpun SJ. Estrogen receptors: Their roles in regulation of vasopressin release for maintenance of fluid and electrolyte homeostasis. Front Neuroendocrinol. (2008) 29:114–27. doi: 10.1016/j.yfrne.2007.08.005 PMC2274006 18022678

[27] Velasquez MT Skelton MM Cowley AW. Water loading and restriction in essential hypertension. Hypertension. (1987) 9:407–14. doi: 10.1161/01.HYP.9.4.407 3549558

[28] Botzki A Rigden DJ Braun S Nukui M Salmen S Hoechstetter J. l-ascorbic acid 6-hexadecanoate, a potent hyaluronidase inhibitor. J Biol Chem. (2004) 279:45990–7. doi: 10.1074/jbc.M406146200 15322107

[29] Jordan J Shannon JR Black BK Ali Y Farley M Costa F. The pressor response to water drinking in humans: A sympathetic reflex? Circulation. (2000) 101:504–9. doi: 10.1161/01.CIR.101.5.504 10662747

[30] Shannon JR Diedrich A Biaggioni I Tank J Robertson RM Robertson D. Water drinking as a treatment for orthostatic syndromes. Am J Med. (2002) 112:355–60. doi: 10.1016/S0002-9343(02)01025-2 11904109

[31] Geelen G Greenleaf JE Keil LC. Drinking-induced plasma vasopressin and norepinephrine changes in dehydrated humans. J Clin Endocrinol Metab. (1996) 81:2131–5. doi: 10.1210/jcem.81.6.8964840 8964840

[32] Callegaro CC Moraes RS Negrão CE Trombetta IC Rondon MU Teixeira MS. Acute water ingestion increases arterial blood pressure in hypertensive and normotensive subjects. J Hum Hypertens. (2007) 21:564–70. doi: 10.1038/sj.jhh.1002188 17344908

[33] Madhavulu B Mohan PR Sreebhushan. RD. Acute effect of excess water intake on blood pressure in healthy Individuals. Asian Pac J Health Sci. (2014) 1:496–9. doi: 10.21276/apjhs

[34] Gibbons CH Schmidt P Biaggioni I Frazier-Mills C Freeman R Isaacson S. The recommendations of a consensus panel for the screening, diagnosis, and treatment of neurogenic orthostatic hypotension and associated supine hypertension. J Neurol. (2017) 264:1567–82. doi: 10.1007/s00415-016-8375-x PMC5533816 28050656

[35] In Sinn D Gibbons CH. Pathophysiology and treatment of orthostatic hypotension in parkinsonian disorders. Curr Treat Options Neurol. (2016) 18:28. doi: 10.1007/s11940-016-0410-9 27138287

[36] Raj SR Biaggioni I Black BK Rali A Jordan J Taneja I. Sodium paradoxically reduces the gastropressor response in patients with orthostatic hypotension. Hypertension. (2006) 48:329–34. doi: 10.1161/01.HYP.0000229906.27330.4f 16785332

[37] Rügheimer L Olerud J Johnsson C Takahashi T Shimizu K Hansell P. Hyaluronan synthases and hyaluronidases in the kidney during changes in hydration status. Matrix Biol. (2009) 28:390–5. doi: 10.1016/j.matbio.2009.07.002 19635555

[38] Göransson V. Renomedullary interstitial cells in culture; the osmolality and oxygen tension influence the extracellular amounts of hyaluronan and cellular expression of CD44. Matrix Biol. (2001) 20:129–36. doi: 10.1016/S0945-053X(01)00129-9 11334714

[39] Ivanova LN Melidi NN. Effects of vasopressin on hyaluronate hydrolase activities and water permeability in the frog urinary bladder. Pflugers Arch. (2001) 443:72–7. doi: 10.1007/s004240100575 11692269

[40] Göransson V Johnsson C Nylander O Hansell P. Renomedullary and intestinal hyaluronan content during body water excess: a study in rats and gerbils. J Physiol. (2002) 542:315–22. doi: 10.1113/jphysiol.2001.014894 PMC2290385 12096072

[41] Stridh S Kerjaschki D Chen Y Rügheimer L Åstrand ABM Johnsson C. Angiotensin converting enzyme inhibition blocks interstitial hyaluronan dissipation in the neonatal rat kidney via hyaluronan synthase 2 and hyaluronidase 1. Matrix Biol. (2011) 30:62–9. doi: 10.1016/j.matbio.2010.09.006 20933085

[42] Fraser JR Laurent TC Laurent UB. Hyaluronan: its nature, distribution, functions and turnover. J Intern Med. (1997) 242:27–33. doi: 10.1046/j.1365-2796.1997.00170.x 9260563

[43] Comper WD Laurent TC. Physiological function of connective tissue polysaccharides. Physiol Rev. (1978) 58:255–315. doi: 10.1152/physrev.1978.58.1.255 414242

[44] Hughes AK Kohan DE. Mechanism of vasopressin-induced contraction of renal medullary interstitial cells. Nephron Physiol. (2006) 103:119–24. doi: 10.1159/000092245 16557030

[45] Dzgoev SG. Selective V2-agonist of vasopressin desmopressin stimulates activity of serum hyaluronidase. Bull Exp Biol Med. (2015) 159:424–6. doi: 10.1007/s10517-015-2981-y 26388582

[46] Grassi D Marraudino M Garcia-Segura LM Panzica GC. The hypothalamic paraventricular nucleus as a central hub for the estrogenic modulation of neuroendocrine function and behavior. Front Neuroendocrinol. (2022) 65:100974. doi: 10.1016/j.yfrne.2021.100974 34995643

[47] Jin X Kim WB Kim MN Jung WW Kang HK Hong EH. Oestrogen inhibits salt-dependent hypertension by suppressing GABAergic excitation in magnocellular AVP neurons. Cardiovasc Res. (2021) 117:2263–74. doi: 10.1093/cvr/cvaa271 PMC10616626 32960965

